# Metal-Free Peptide Semiconductor-Enhanced Raman Scattering

**DOI:** 10.1021/acs.nanolett.4c04049

**Published:** 2024-12-11

**Authors:** Sawsan Almohammed, Agata Fularz, Ahmed Alanazi, Mohammed Benali Kanoun, Souraya Goumri Said, Kai Tao, Brian J. Rodriguez, James H. Rice

**Affiliations:** † School of Physics, University College Dublin, Belfield, Dublin 4, Ireland; ‡ Conway Institute of Biomolecular and Biomedical Research, University College Dublin, Belfield, Dublin 4, Ireland; § Department of Mathematics and Sciences, College of Humanities and Sciences, Prince Sultan University, P.O. Box 66833, Riyadh 11586, Saudi Arabia; ∥ College of Science and General Studies, Physics Department, Alfaisal University, P.O. Box 50927, Riyadh 11533, Saudi Arabia; ⊥ State Key Laboratory of Fluid Power and Mechatronic Systems, Key Laboratory of Advanced Manufacturing Technology of Zhejiang Province, School of Mechanical Engineering, Zhejiang University, Hangzhou 310058, China

**Keywords:** Bioinspired materials, peptides, chemical detection, mechanism, Raman

## Abstract

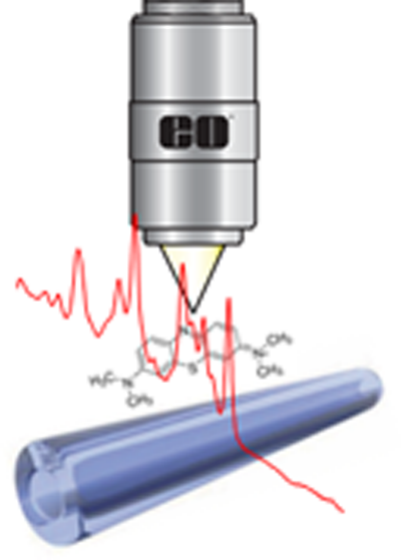

There is a growing demand for sustainable and safe materials in developing technological systems and devices, including those that enhance Raman scattering. Organic (bio) materials based on simple peptides are one class of such materials. This study investigates self-assembled semiconducting peptides as metal-free substrates for surface-enhanced Raman scattering. Our results reveal significant variations in Raman enhancement factors, spanning up to 2 orders of magnitude. We examined specific Raman enhancement selection rules related to the energy levels and structural configurations of the probe molecules. The effectiveness of these rules underscores the importance of strong molecule-peptide coupling and efficient charge transfer for achieving optimal Raman enhancement factors. These insights offer a foundational understanding of peptide–molecule interactions and the underlying chemical mechanisms driving Raman enhancement, highlighting the potential of organic semiconductor-based materials as highly effective platforms for enhancing Raman scattering in chemical sensing applications.

Surface-enhanced Raman scattering (SERS) spectroscopy is a powerful analytical technique known for its rapid, label-free, and highly sensitive molecular fingerprinting capabilities.^1,2^ It finds applications in various fields including optoelectronic device characterization, chemical sensors, and medical diagnostics.^2,3^ The most commonly used SERS substrate materials are plasmonic nanostructured metals such as Au and Ag, which enhance SERS through the electromagnetic mechanism (EM), resulting in significant signal amplification.^4^ This mechanism involves concentrating electromagnetic fields near nanostructured conducting materials like Ag and Au and is achieved through the excitation of localized surface-plasmon resonances in proximity to target molecules.

While metals dominate SERS substrate design, there is growing interest in semiconductor materials due to their advantages such as sustainability, flexibility, and biocompatibility.^4,5^ Semiconductor-based SERS operates via the chemical enhancement (CE) mechanism, which alters the polarizability of probe molecules through charge-transfer transitions between the molecule and substrate.^6^ Despite these advantages, EM remains the primary enhancement mechanism in SERS, capable of enhancing Raman signals by factors as high as 10^10^, compared to typically lower enhancement factors for CE.^7^

Various materials, including metal oxides such as ZnO, TiO_2_, and single-element semiconductors like graphene and Si, have shown capability in enhancing Raman scattering through CE.^7−9^ Graphene, in particular, has garnered significant research attention for its ability to enhance Raman signals by orders of magnitude, varying significantly depending on the molecules involved.^10,11^ This variability stems from differences in the energy levels and structural characteristics of the probe molecules.^10,12^ Effective enhancement requires optimal alignment of molecular energy levels, specifically the highest occupied molecular orbital (HOMO) and lowest unoccupied molecular orbital (LUMO), with the Fermi level of the active substrate.^10,13^ The molecular structure also plays a crucial role, as molecules with symmetry resembling that of graphene can achieve strong coupling and efficient charge transfer with the substrate, thereby enhancing Raman scattering effectively. Utilizing graphene as a substrate for Raman enhancement has shown potential to produce robust and reproducible Raman signals, with enhancement factors approaching 100.^10,13^

There is a growing demand for sustainable and safe materials in developing technological systems and devices, including those that enhance Raman scattering. Bioinspired materials like peptides, (in addition to graphene), are noteworthy in this regard. Peptide self-assembly is particularly attractive for creating precisely defined nanostructures through the coordinated self-association of peptides or their derivatives via noncovalent interactions.^14^ Peptides offer inherent advantages such as easy synthesis, thermal and chemical stability, well-defined molecular structures, and excellent assembling properties. These qualities make self-assembling peptides appealing as scaffolds to mimic natural self-assembly processes and create complex structures or functional nanomaterials with high precision.^15^

Short peptide motifs have garnered significant interest for their ability to form supramolecular structures or nanomaterials due to their straightforward synthesis and remarkable self-assembling properties. Additionally, they can demonstrate piezoelectric and pyroelectric properties.^15^ Diphenylalanine, a short-chain peptide derived from the natural amino acid phenylalanine, can self-assemble into micro and nanoscale tubular or wire structures.^15,16^ Studies have demonstrated that combining diphenylalanine peptides with plasmon active metal nanostructures can significantly enhance Raman scattering performance compared to using plasmon nanostructures alone.^17,18^ Moreover, diphenylalanine-metal heteromaterials have shown synergistic electro-optical properties under an external electric field.^19,20^ By adjusting diphenylalanine’s density of states from semiconductor-like to metallic, efficient charge transfer from the nanotube to metal nanoparticles enhances surface-enhanced Raman scattering signals for a variety of analyte molecules.^19^

Here, we explore the potential of peptides beyond diphenylalanine to enhance Raman scattering signals from probe molecules without the presence of metal. We investigate a range of peptides capable of self-assembling into nanofiber-like nanostructures and interacting with probe molecules. Our findings reveal strong molecular selectivity for peptide-enhanced Raman scattering (PSERS), with enhancement factors (EF) varying significantly among different probe molecules by up to 2 orders of magnitude. This variation in EFs is explained through the specific Raman enhancement selection rules associated with the energy levels and structural configurations of probe molecules. Our results offer fundamental insights into peptide-molecule interactions and the chemical mechanism behind Raman enhancement, demonstrating that peptide-based semiconductor materials can serve as highly efficient platforms for enhanced Raman scattering in molecular sensing applications.

A range of peptides (listed in Figure S1, Supporting Information) were prepared with nanowire-like aspect ratios. The structures formed by each peptide were analyzed using scanning electron microscopy (SEM). SEM images (Figure 1 and Figure S2, Supporting Information) captured the self-assembled structures of nine peptides. Among these were diphenylalanine (Phe-Phe) and ditryptophan (Trp-Trp), two widely studied short-chain peptides. It is known that Phe-Phe self-assembles into nanotubes^14−16^ while Trp-Trp forms nanowire assemblies, as shown in SEM images (Figure 1, Figure S2, Supporting Information). These nanowires result from tryptophan’s strong quadrupole interactions, which facilitate stacking and the formation of stable structures.^21^ Tryptophan-based peptide networks have been reported to show superior electron transfer and conductivity properties compared to phenylalanine-based analogues, potentially benefiting CE Raman enhancement processes.^22^ In addition to Phe-Phe and Trp-Trp, seven other peptides were studied (Table S1, Supporting Information), including Cyclo-Trp-Tyr, Cyclo-Trp-(d)Trp, Cyclo-Gly-Trp, Trp-Phe, Trp-(d)Trp, Cyclo-Phe-Trp, and Cyclo-Trp-Trp. SEM imaging shows that these peptides also form nanotube- or nanowire-like structures (Figure 1, and Figure S2, Supporting Information).

**Figure 1:**
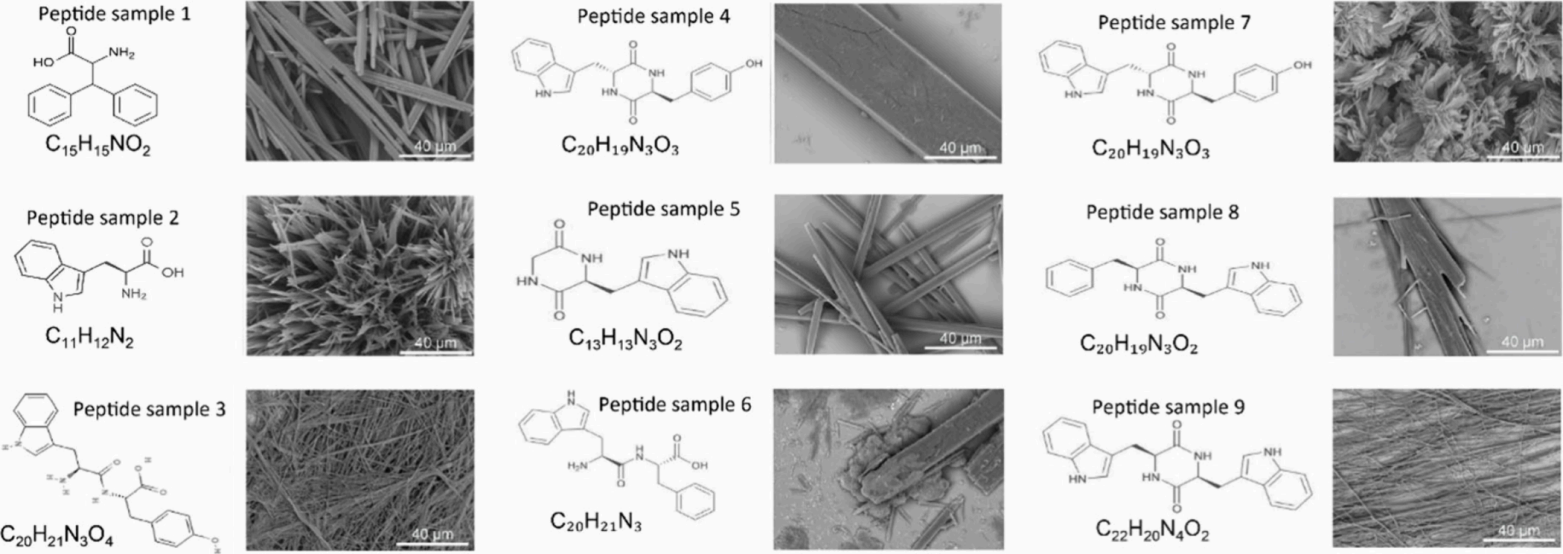
Scanning electron microscopy (SEM) images and chemical formula of the nine self-assembled peptides studied. The abbreviated name for each peptide is shown along with the SEM image as also outlined additionally in Figure S1, Supporting Information.

To understand the electronic structure of each peptide nanostructure, the optical absorption spectra of the peptides were recorded. These spectra were analyzed using Tauc plots^23^ (Supporting Information, Figure S3) to determine the peptide’s band gap (*E*_g_). Each peptide has a distinct band gap value; for example, Phe-Phe has an *E*_g_ of 3.97 ± 0.4 eV, while Trp-Phe has an *E*_g_ of 2.83 ± 0.01 eV.

Kelvin probe force microscopy (KPFM) was then applied to further characterize the peptides. KPFM imaging measured the surface potential and work function (see Figure 2a, and Figure S4 in the Supporting Information). Each peptide type possesses a unique surface potential. For instance, Cyclo-Gly-Trp has a surface potential of 577 ± 18 mV, whereas Trp-(*d*)-Trp has a surface potential of 112 ± 87 mV. Additionally, every peptide type possesses a distinct Fermi level, calculated from Kelvin probe and optical absorption measurements (Supporting Information, Figures S3 and S4).^24^ The Fermi level (*E*_F_) varies depending on the peptide, with *E*_F_ = 4.93 ± 0.06 eV for Cyclo-Gly-Trp and *E*_F_ = 5.62 ± 0.08 eV for Trp-(*d*)-Trp.

**Figure 2:**
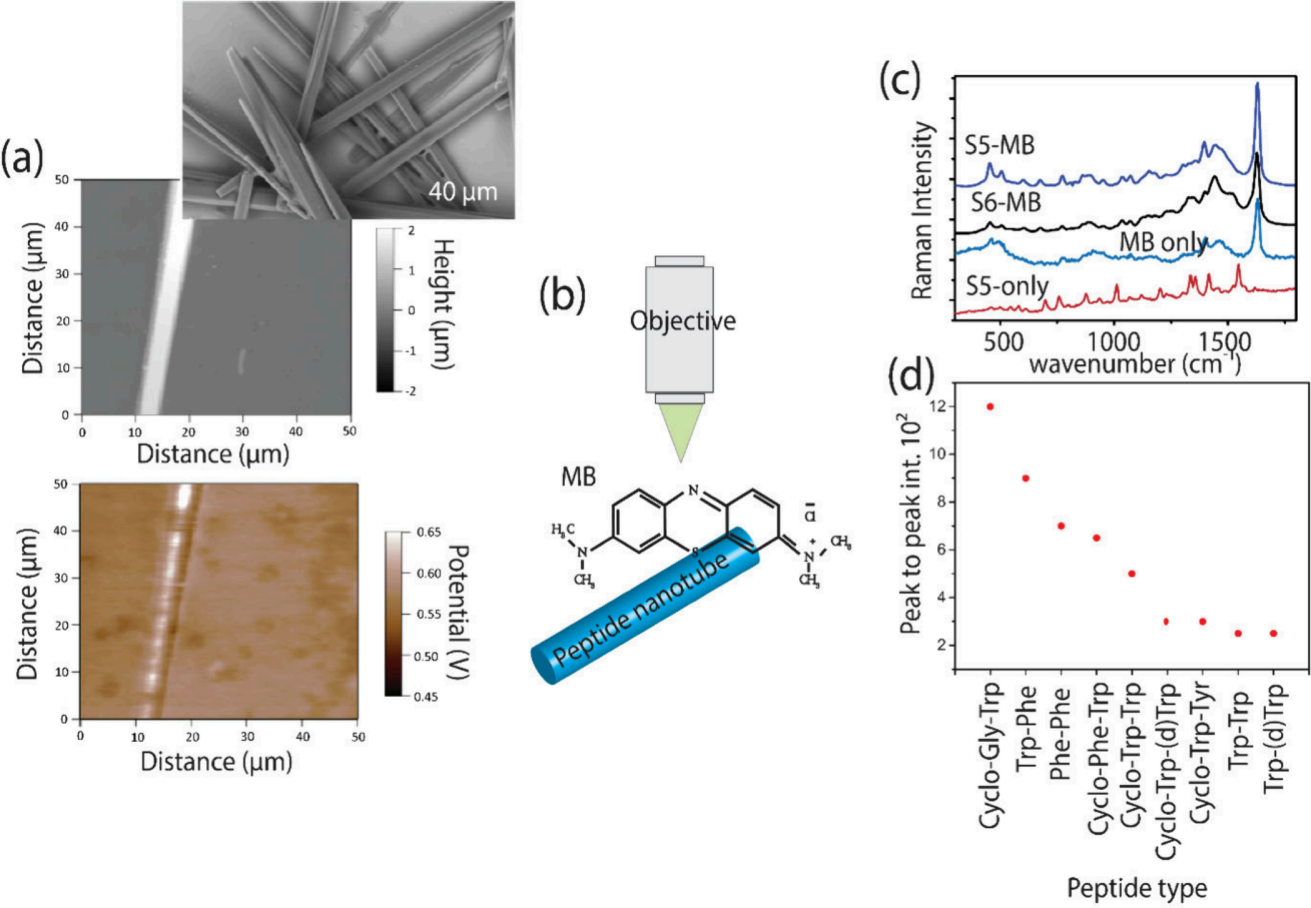
Kelvin probe and PSERS spectral data. (a) Kelvin probe microscopy potential images for a single Cyclo-Gly-Trp nanotube or nanowire-like structures (bottom) are shown with an AFM topography image (top). A SEM image of the nanotube or nanowire-like structure is shown above the AFM image (b) Schematic drawing showing the laser irradiation and resulting PSERS from Cyclo-Gly-Trp, with a MB molecule deposition upon the peptide. (c) PSERS of MB on Cyclo-Gly-Trp and Trp-Phe along with the Raman spectra for the probe molecule MB and the peptide Cyclo-Gly-Trp only. (d) Plot of PSERS signal intensity for MB on each peptide.

The peptides were then studied for their potential to enhance Raman scattering cross sections from absorbed analyte molecules. Peptide semiconductor-enhanced Raman scattering (PSERS) using methylene blue (MB, schematically shown in Figure 2b) at a concentration of 10^–5^ M was evaluated across nine different peptide types. PSERS peaks for MB on each peptide (Figure 2c, and Figure S5 in the Supporting Information) were visible and detectable. Characteristic bands included ν(C–C) ring stretching at 1560 cm^–1^, ν(C–N) symmetric and asymmetric stretching at 1433 cm^–1^, and the δ(C–N–C) skeletal deformation mode at 448–500 cm^–1^, all of which matched the literature values.^25−27^ Comparing the PSERS intensities from MB on each peptide type revealed variations in MB Raman signal intensities (Figure 2d). The peptide Cyclo-Gly-Trp showed the highest MB PSERS signal intensity, while Trp-(d)Trp showed the lowest. The variation in Raman enhancement, assessed through the peak-to-peak intensity of the 1535 cm^–1^ band, showed that the MB signal varied by approximately an order of magnitude when absorbed onto Cyclo-Gly-Trp relative to Cyclo-Trp-(*d*)Trp or tryptophan-(*d*)-tryptophan (Trp-(*d*)Trp).

The morphology of the peptide nanostructures may explain the variations in Raman enhancement for MB. The morphology of Cyclo-Gly-Trp, which supports the highest Raman signal intensity for MB, is very similar to that of Cyclo-Trp-(*d*)Trp, which supports a much lower (>10-fold) enhancement in Raman signal. Both peptides self-assemble into nanostructures with similar aspect ratios of width/length (Figure 1, and Figure S2 in the Supporting Information). Cyclo-Gly-Trp has an average diameter of 15 μm, while Cyclo-Trp-(*d*)Trp has an average tube diameter of 12 μm. Peptides with smaller diameters offer a larger surface area, which can absorb more analyte molecules and potentially produce a higher PSERS signal enhancement. Cyclo-Trp-Tyr, for example, produces structures with much smaller diameters (∼750 nm) relative to Cyclo-Gly-Trp but shows a lower PSERS signal for MB. This suggests that peptide morphology alone does not predict PSERS potential. Studies on graphene-enhanced Raman scattering have shown that the interaction strength between graphene and the probe molecule depends primarily on the degree of matching between the molecular energy levels and symmetries with those of graphene.^7,10^

To assess the impact of probe molecular energy levels and molecular symmetries on PSERS from Cyclo-Gly-TrP, we examined a series of molecules beyond MB. The PSERS spectra and EF from five molecules, MB, TMPyP, 4ABT, NIP, and AMP (molecular abbreviations defined in Figure S6, Supporting Information), on Cyclo-Gly-TrP were recorded. We first analyzed the PSERS EF for MB and TMPyP. The EF values for MB (at 1590 cm^–1^) and TMPyP (at 1620 cm^–1^) were measured as 24 and 29, respectively, following the methodology described in the Supporting Information methods (Figure 3a).

**Figure 3:**
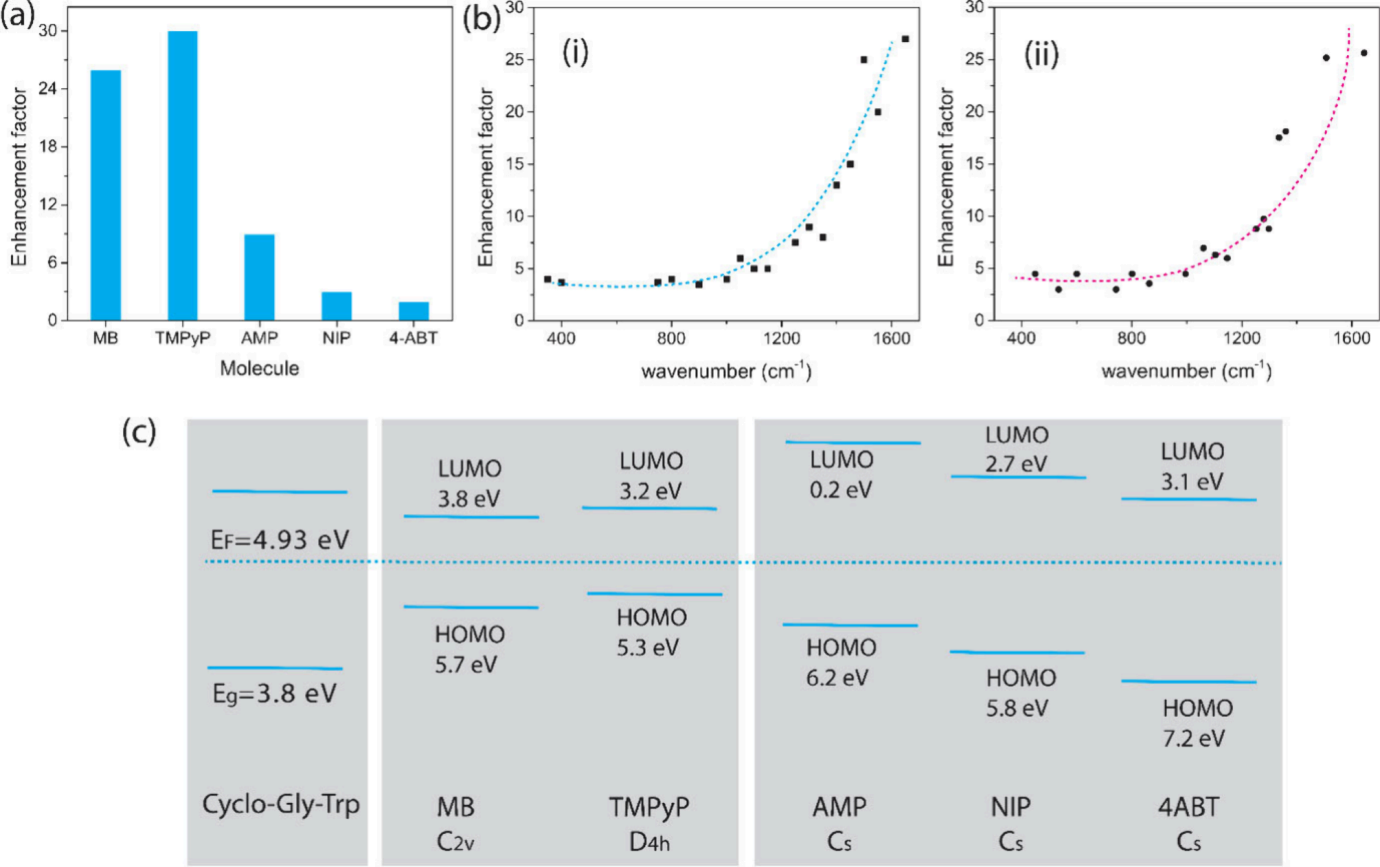
PSERS data for molecules on Cyclo-Gly-Trp. (a) Plot of calculated EF for different molecules on Cyclo-Gly-Trp. (b) EF as a function of phonon frequency obtained for (i) MB and (ii) TMPyP. (c) Energy level diagram for the peptide Cyclo-Gly-Trp and molecules. The peptides Fermi energy (*E*_F_) being calculated from AFM/Kelvin probe measurements as outlined in Figure S4, Supporting Information.

We then plotted the EF of MB and TMPyP on Cyclo-Gly-TrP against phonon frequency (Figure 3b(i,ii)), revealing that as the phonon frequency increases from 500 cm^–1^ (0.05 eV) to 1600 cm^–1^ (0.2 eV), EF also increases. Studies of EF for graphene-enhanced Raman scattering have shown strong Raman enhancement when the phonon energy approximates the energy difference between the Fermi level of graphene and the HOMO/LUMO levels of the molecules.^28,29^ According to perturbation theory and Fermi’s Golden Rule, Raman scattering efficiency is inversely proportional to the energy difference between the semiconductor Fermi level, phonon energy, and the electronic levels of the molecule.^28^ Thus, EF increases when the phonon energy matches either the energy difference between the Fermi level and the molecular HOMO/LUMO levels or the energy difference between the laser energy and the HOMO–LUMO gap.

The molecules TMPyP and MB have similar HOMO/LUMO levels at 5.7/3.8 eV and 5.5/3.3 eV, respectively (Figure 3c).^30,31^ The laser energy (λ_ex_ = 2.3 eV) closely aligns with the HOMO–LUMO energy gaps of MB (1.9 eV) and TMPyP (2.2 eV), facilitating a strong Raman resonance condition. Additionally, the energy difference between the Cyclo-Gly-TrP Fermi level (*E*_F_ = 4.93 eV) and the electronic levels of MB and TMPyP is similar, with Δ*E* separation <1.8 eV, leading to similar PSERS EF enhancements for both. This enhancement similarity is further supported by the molecular structures, as both TMPyP and MB are highly symmetric, with D4h and C2v point groups, respectively. These symmetries allow for efficient physical contact with the peptide surface.

We then examined PSERS using Cyclo-Gly-TrP with molecules that possessed lower symmetry and a larger range of molecular energy levels, specifically AMP, NIP, and 4ABT. Comparing the PSERS EF for MB or TMPyP to AMP shows that the AMP EF is much weaker, by a factor of >3 (Figure 3a). This low EF can be explained in part by the reduced symmetry of AMP, which has a C_s_ point group. This reduces the potential for effective contact between the molecule and peptide surface, making it relatively challenging to facilitate charge transfer between the peptide and molecule.

While these molecules 4ABT, NIP, and AMP have the same molecular structure (Cs symmetry), their HOMO/LUMO energies differ: −5.0/–1.2 eV for AMP, −4.0/–7.2 eV for 4ABT, and −5.6/–9.4 eV for NIP (Figure 3c).^32−34^ Raman enhancement was observed for all three molecules on Cyclo-Gly-TrP, but with different EFs (Figure 3a). The EF from AMP was 7.2, compared to 1.7 for NIP and 1.5 for 4ABT. The EF from AMP is 4.2 times higher than the EF from NIP. These results indicate that despite their similar molecular structures, the presence of different molecular HOMO/LUMO levels plays an important role in PSERS enhancement.

The PSERS EF and HOMO energy levels decrease when comparing AMP (6.2 eV, EF = 7.2) to NIP (6.8 eV, EF = 1.7) and 4ABT (7.2 eV, EF = 1.5). The PSERS EFs of the molecules fall as the HOMO (and corresponding LUMO) energy levels move farther energetically from the peptide’s Cyclo-Gly-TrP Fermi energy level. This is in line with Fermi’s Golden Rule, where a reduction in HOMO energy levels increases the energy difference between the peptide’s Fermi level and the electronic levels of the molecule.^10^ Stronger PSERS enhancement for NIP was observed when changing the peptide. PSERS spectra for NIP using the peptide Trp-Phe showed the highest signal (Figure 4a). The EF was measured to be 6.8 for the Trp-Phe peptide, compared to an EF of 1.7 for Cyclo-Gly-TrP. These EF values are due to the Fermi energy level for Trp-Phe (*E*_F_ = 5.16 eV) being higher in energy compared to Cyclo-Gly-TrP (*E*_F_ = 4.93 eV). This results in stronger resonance with the HOMO/LUMO levels, leading to stronger PSERS enhancement for NIP on Trp-Phe compared to Cyclo-Gly-TrP.

**Figure 4:**
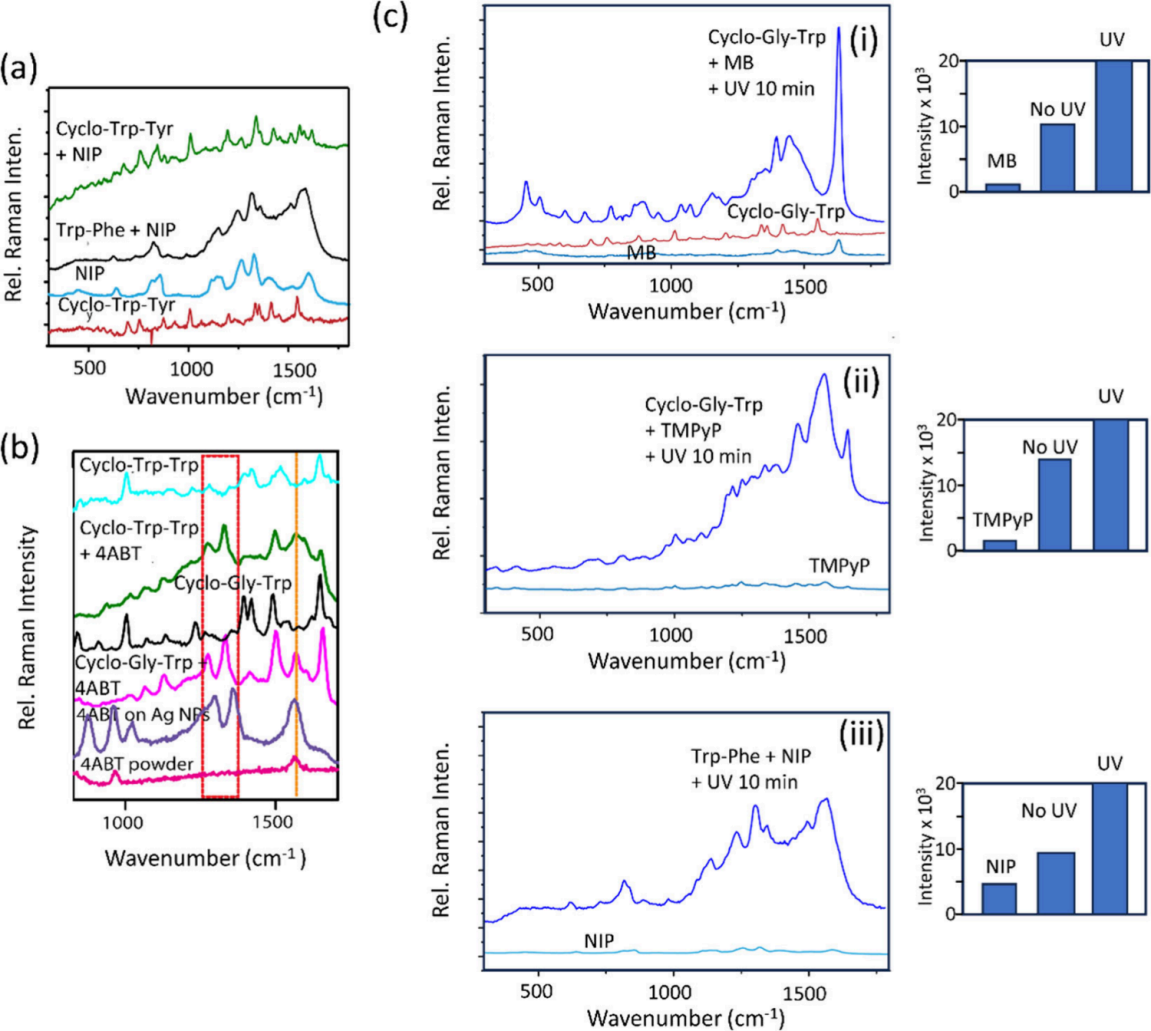
(a,b) PSERS spectra for NIP and 4ABT on different peptides, shown along with the Raman spectra for the molecule and peptides. (c) PSERS spectra recorded for three different molecules (i–iii) on Cyclo-Gly-TrP, before and following superband gap irradiation (*E*_irradation_ = 4.6 eV) for 10 min. Shown on the right are histogram plots of PSERS signal intensity for the molecule on silicon and peptide before and after irradiation.

To assess in more depth how the molecule and peptide interact, DFT (density functional theory) simulations were conducted. DFT simulations for Cyclo-Gly-Trp, with and without absorbed MB molecules, were performed (Supporting Information, Figure S7). DFT (density functional theory) simulations were conducted, examining Cyclo-Gly-Trp both with and without absorbed MB molecules.

Analysis of the DOS plot indicates that the adsorption of MB onto the peptide introduces molecule-originated electronic states inside the peptide’s band gap region. Compared to the peptide in the absence of MB, the valence and conduction bands of MB on Cyclo-Gly-Trp shift toward lower energies. The occupied molecular states emerge inside the band gap upon the adsorption of the MB molecule. The interaction between MB and the peptide not only alters the band edges of both the valence and conduction bands but also forms energy states within the band gap. The charge density plot of MB adsorbed on Cyclo-Gly-Trp (Supporting Information, Figure S8) shows an overlap between the charge densities of MB and the 2p orbitals of Cyclo-Gly-Trp indicating electrostatic interaction facilitating charge transfer between the two moieties.

Additional DFT studies of Phe-Phe with MB were also performed (Supporting Information, Figure S9) as Phe-Phe with MB also showed a strong SERS signal enhancement (EF = 15) (Figure 2d). The DOS plot indicates that the adsorption of MB onto the peptide introduces molecule-originated electronic states inside the peptide’s band gap region, like MB on Cyclo-Gly-Trp. Optical absorption spectral measurements for MB on the peptide (Supporting Information, Figure S9) showed a redshift of approximately 20 nm for the band located at 615 nm, near the valence band edge. These absorption changes demonstrate strong molecule–peptide interactions, lowering the valence and conduction band edges in line with the DOS simulations.

We then examined the PSERS spectra for 4ABT on a series of peptides (Figure 4b, and Figure S10 in Supporting Information). The PSERS spectra for 4ABT on the peptides Cyclo-Gly-Trp and Cyclo-Trp-Trp show strong Raman peaks arising from 4ABT at 1430, 1385, and 1570 cm^–1^ (Figure 4b). The SERS spectrum for 4ABT recorded on silver nanoparticles (Figure 4b) shows very similar spectral features, with strong Raman bands at 1430, 1385, and 1570 cm^–1^. The SERS spectra of 4ABT are reported to arise from the dimerization of 4ABT molecules to form DMAB (dimercaptoazobenzene), giving rise to different Raman peaks compared to the Raman spectrum of 4ABT on silicon.^35,36^ This suggests that the dimerization of 4ABT occurs on the peptide in the absence of silver nanoparticles. The mechanism reported for dimerization on silver nanoparticles involves thermally mediated charge transfer, creating singlet oxygen,^37^ where electrons from localized surface plasmons are excited and support charge-mediated singlet oxygen production. In contrast, a different mechanism is at play for charge-mediated singlet oxygen production of 4ABT on peptides, as peptides do not support surface plasmon resonances.

The piezoelectric and pyroelectric properties of the peptides can potential influence the mechanism for dimerization of 4ABT. Cyclo-Gly-Trp and Cyclo-Trp-Trp are reported to be piezoelectric and pyroelectric.^38,39^ Cyclo-Trp-Trp and Cyclo-Gly-Trp are reported to have significant piezoelectric coefficients of 14 pC/N and 54 pC/N, respectively,^38,39^ which are higher than traditional inorganic piezoelectric materials such as ZnO (d_33_ = 12 pC/N) and CdS (d_15_ = 12 pC/N).^38^ The pyroelectric coefficient of Cyclo-Trp-Trp has been measured to be 35 × 10^–6^ Cm^–2^K^–1^,^39^ which is comparable to the well-known piezoelectric/pyroelectric polymer polyvinylidene fluoride (PVDF) (−0.27 × 10^–4^ Cm^–2^K^–1^).^40^ The pyroelectric potential of the peptide is potentially activated by the localized heating from the Raman excitation laser, giving rise to an electrical potential, where charge can transfer to form singlet oxygen, leading to the dimerization of 4ABT and creating DMAB. This indicates that the pyroelectric potential may be an additional parameter at play, in addition to energy levels and molecular symmetry.

PSERS EF can be significantly improved using superband gap irradiation of the sample (energy = 4.6 eV) (Figure 4c). UV-irradiated MB on Cyclo-Gly-Trp showed approximately a 2-fold increase in the PSERS signal, compared to TMPyP on Cyclo-Gly-Trp, which was enhanced by approximately 1.5 times (Figure 4c). The PSERS signal from NIP on Cyclo-Gly-Trp was enhanced by a factor of about 2.5 following UV irradiation (Figure 4c). This demonstrates the stability of the peptide substrate and the ability to enhance the PSERS EF via superband gap irradiation. Studies using UV irradiation to enhance SERS signals with diphenylene peptide substrates (with silver nanoparticles) have been reported.^41^ This study found that the thermal conductivity of the diphenylene peptide nanotubes prevents the photodegradation of probe molecules, likely by acting as a heat sink during UV irradiation. It was proposed that the mechanism for the increased SERS signal resulted from the charge transfer of photogenerated electrons from the peptides (during UV irradiation) to the silver nanoparticles. In the absence of silver nanoparticles, the photogenerated electrons can potentially transferred to the molecule, enhancing the PSERS signal strength through increased dipole strength, leading to a stronger Raman cross-section.

Our results demonstrate significant molecular selectivity in the PSERS effect, with enhancement factors displaying notable variations—spanning up to 2 orders of magnitude, among different probe molecules. We identify two key selection rules that contribute to the Raman enhancement factor. One factor is related to how well the molecular energy levels of the probe molecules match those of the peptides. Strong charge-transfer interactions between the molecule and peptide occur when their energy levels are well-matched, resulting in strong PSERS.

A second factor relates to the structural symmetry of the probe molecule, as defined by its point symmetry. Strong molecule-peptide coupling occurs when the molecule can effectively contact the peptide. The point group of the molecule is indicative of the probe’s symmetry and is related to its potential to interface with the peptide’s surface.

We also outline the potential contribution of pyroelectric potential from the peptide nanomaterials in enhancing PSERS signals, where local heat from the Raman excitation laser potentially activates a pyroelectric potential in the peptide.

These findings offer crucial insights into the interactions between piezoelectric peptides and molecules, as well as the chemical processes driving Raman enhancement. Moreover, they highlight the potential of organic semiconductor-based materials as exceptionally effective mediums for boosting Raman scattering in chemical sensing device design.

## Supporting Information Available

SEM, AFM, Kelvin probe, and Raman measurements; Ccalculated band structure, and electron density difference with supporting UV/vis experimental data; additional details about DFT calculations, sample preparation, and Raman measurements (PDF)
