# Supplementary material for: Metal-Free Peptide Semiconductor-Enhanced Raman Scattering
Source: Nano Lett. 2024 Dec 11;24(51):16301–8. doi: 10.1021/acs.nanolett.4c04049 (PMC11673574; doi:10.1021/acs.nanolett.4c04049)
Supplement: Supplementary file 1 — nl4c04049_si_001.pdf [file nl4c04049_si_001.pdf]

Metal-Free Peptide Semiconductor-Enhanced Raman Scattering

Sawsan Almohammed [a, b], Agata Fularz [a], Ahmed Alanazi [a], Mohammed Benali Kanoun[c], Souraya Goumri Said[d], Kai Tao[e], Brian J. Rodriguez * [a,b], and James H. Rice * [a]

[a]*School of Physics, University College Dublin, Belfield, Dublin 4, Ireland*
[b]*Conway Institute of Biomolecular and Biomedical Research, University College Dublin, Belfield, Dublin 4, Ireland*
[c]*Department of Mathematics and Sciences, College of Humanities and Sciences, Prince Sultan University,* P.O. Box 66833, Riyadh 11586, *Saudi Arabia*
[d]*College of Science and General Studies, Physics Department, Alfaisal University, P.O. Box 50927, Riyadh, 11533, Saudi Arabia*
[e]*State Key Laboratory of Fluid Power and Mechatronic Systems, Key Laboratory of Advanced Manufacturing Technology of Zhejiang Province, School of Mechanical Engineering, Zhejiang University, Hangzhou 310058, China*

Supplementary information

Section 1 additional figures
Section 2 methods

Section 1 additional figures

sample	
**No. 1**: diphenylalanine (**Phe-Phe**)	
**No. 2**: ditryptophan (**Trp-Trp**)	
**No. 3**: cyclo-tryptophan-tyrosine (**Cyclo-Trp-Tyr**)	
**No. 4**: cyclo-tryptophan-(d)-tryptophan (**Cyclo-Trp-(d)Trp**)	
**No. 5**: cyclo-glycine-tryptophan (**Cyclo-Gly-Trp**)	
**No. 6**: tryptophan-phenylalanine (**Trp-Phe**)	
**No. 7**: tryptophan-(d)-tryptophan (**Trp-(d)Trp**)	
**No. 8**: cyclo-phenylalanine-tryptophan (**Cyclo-Phe-Trp**)	
**No. 9**: cyclo-tryptophan-tryptophan (**Cyclo-Trp-Trp**)	

Fig S1. Table outlining the abbreviations for each peptide studied.

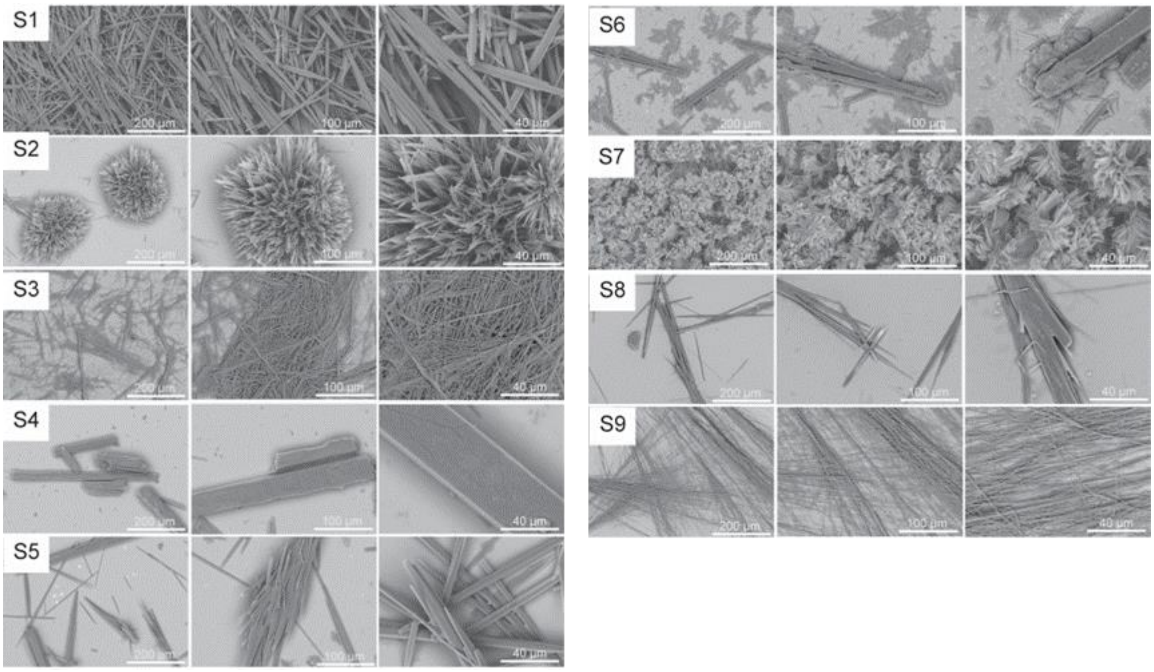

Fig S2. Scanning electron microscopy (SEM) of each peptide recorded at three different magnifications. Shown also is a table of peptide names and abbreviations for reference.

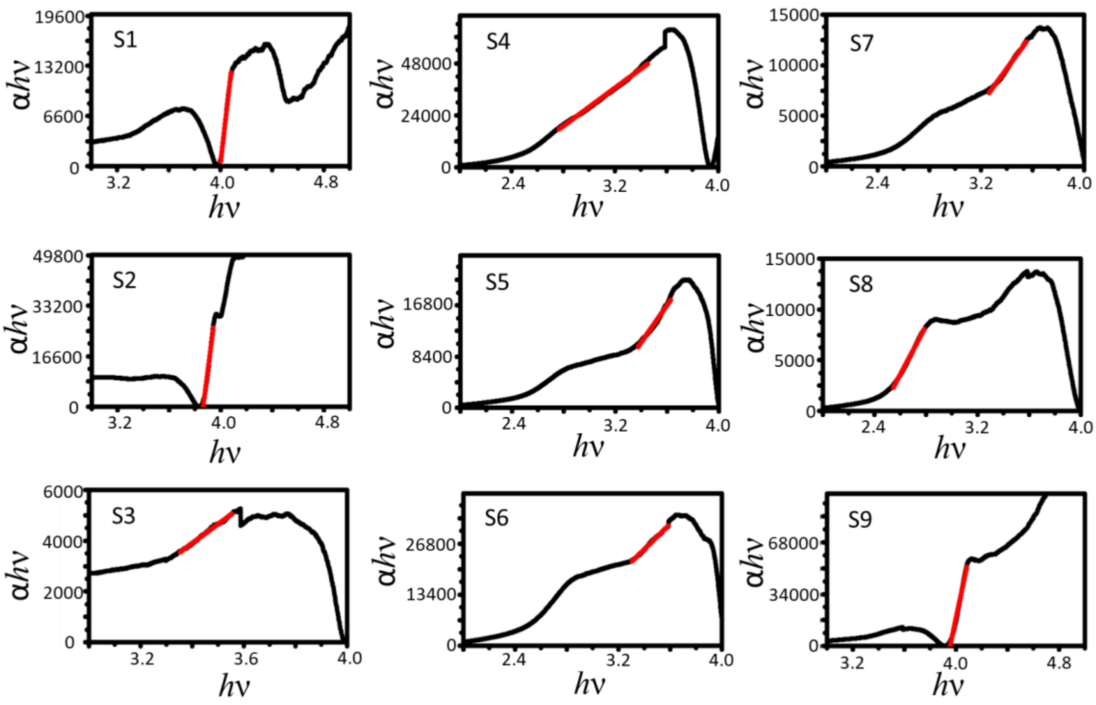

Sample	Without silver
S1	3.97 ± 0.04 eV
S2	3.13 ± 0.02 eV
S3	3.84 ± 0.11 eV
S4	3.14 ± 0.02 eV
S5	3.22 ± 0.01 eV
S6	2.83 ± 0.01 eV
S7	2.77 ± 0.01 eV
S8	3.88 ± 0.05 eV
S9	3.11 ± 0.02 eV

Fig S3. Optical absorption spectra were recorded for each peptide on a coverslip. The table shows the calculated band gap via Tau plots, shown as red lines.

Average	Surface potential on tubes (mV)	Surface potential on background (mV)	Work function on tubes (eV)	Work function on background (eV)	Fermi level (eV)
Standard sample			Assume tip work function is ~5.5 eV	Sample workfunction $=W_{tip}$ –surface potential	
Sample 1	114.5+/-239.1	69.4+/-125.1	5.39+/-0.24	5.43+/-0.13	5.39 eV
Sample 3	90.6+/-83.9	80.1+/-71.4	5.41+/-0.08	5.42+/-0.07	5.41 eV
Sample 4	256.2+/-79.6	243.07+/-85.4	5.24+/-0.08	5.26+/-0.09	5.24 eV
Sample 5	576.8+/-18.3	564.2+/-7.7	4.93+/-0.02	4.94+/-0.01	4.93 eV
Sample 6	357.9+/-17.8	271.7+/-18.3	5.16+/-0.02	5.23+/-0.02	5.16 eV
Sample 7	-122.3+/-87.3	-142.5+/-53.0	5.62+/-0.08	5.64+/-0.05	5.62 eV
Sample 8	154.8+/-21.5	138.73+/-33.3	5.35+/-0.02	5.36+/-0.03	5.35 eV
Sample 9	264.8+/-35.5	243.9+/-52.3	5.24+/-0.04	5.26+/-0.05	5.25 eV

Fig S4. Kelvin probe measurements of the peptides. Outlined in the table are surface potential, work function and Fermi energy level values.

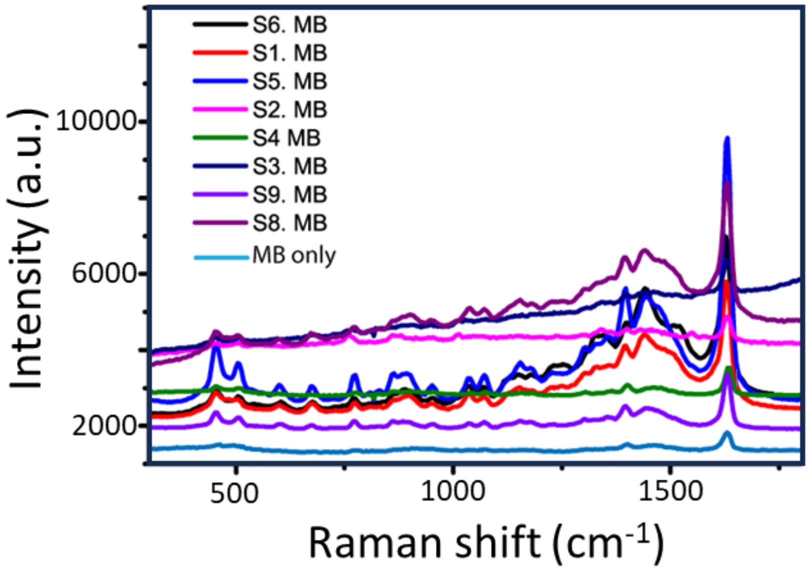

Fig S5. Peptide semiconductor-enhanced Raman scattering (PSERS) using methylene blue (MB) at a concentration of 10^{-5} M recorded for different peptide types.

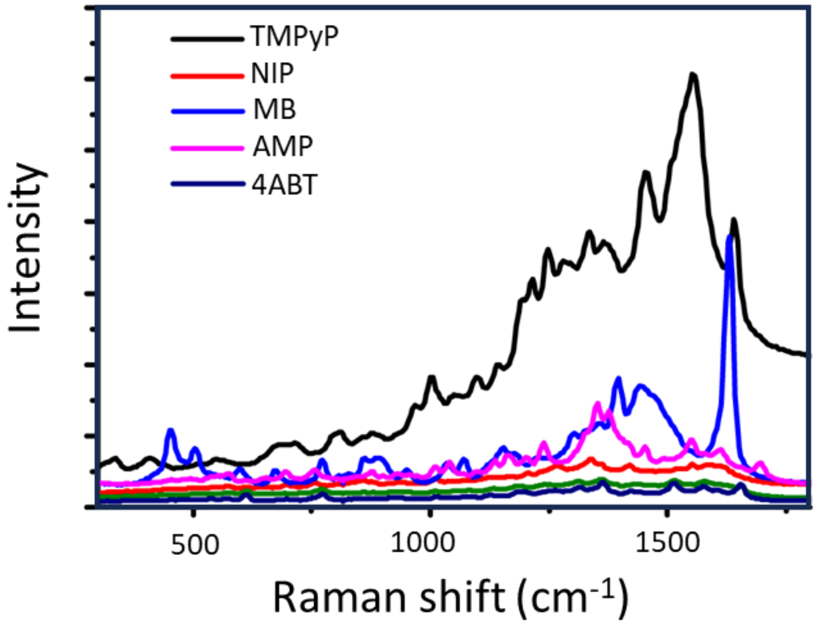

Fig S6. Peptide semiconductor-enhanced Raman scattering (PSERS) using Cyclo-Gly-Trp as the peptide with a range of probe molecules. Specifically MB (Methylene Blue), TMPyP (Tetrakis(1-methyl-4-pyridinio)porphyrin-tetra(p-toluenesulfonate)), 4ABT (4-aminothiophenol), NIP (2-nitrophenol) and AMP (2-aminophenol) at a concentration of 10^{-5} M.

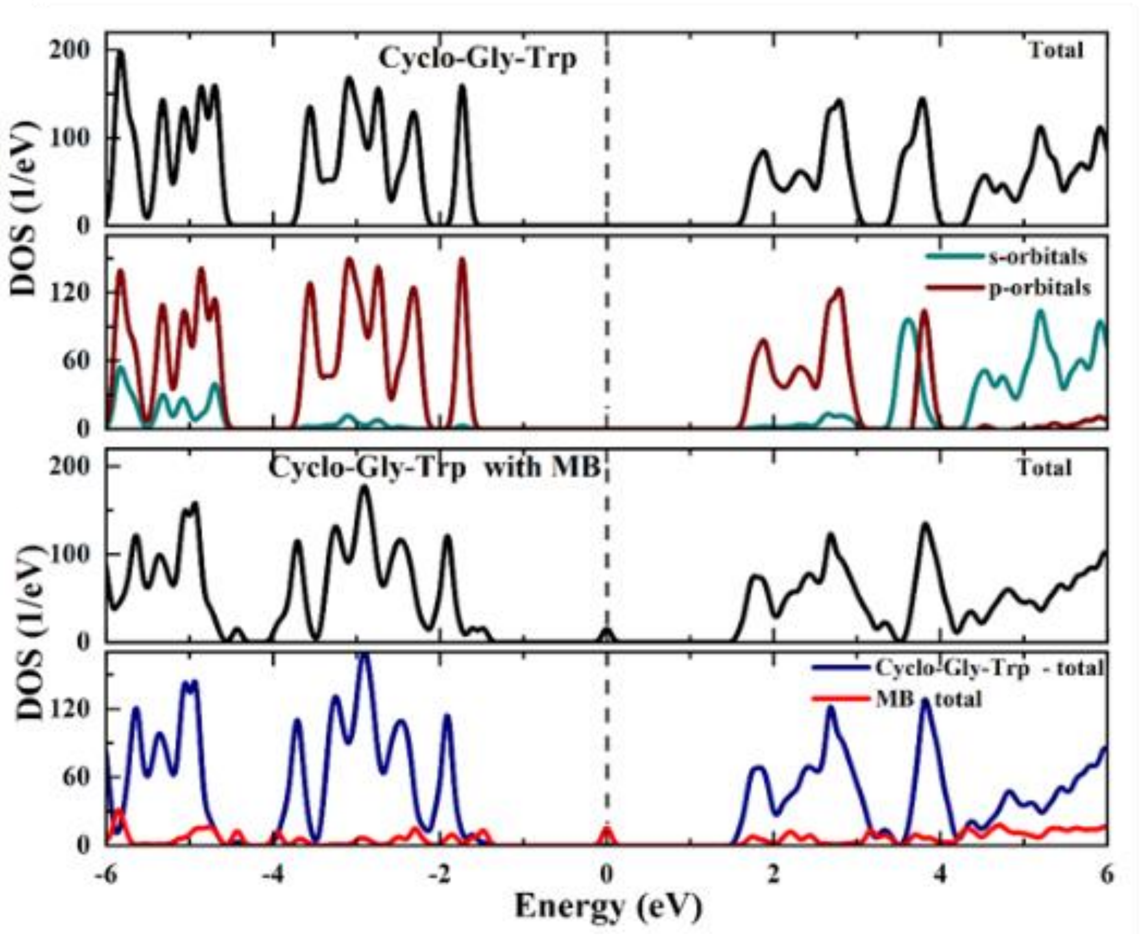

S7. Calculated total and projected DOS for of MB on Cyclo-Gly-Trp. (i) Cyclo-Gly-Trp only (ii) MB on Cyclo-Gly-Trp. The Fermi level is centred at zero. Compared to the peptide in the absence of MB, the valence and conduction bands of MB on Cyclo-Gly-Trp shift toward lower energies. The interaction between MB and the peptide not only alters the band edges of both the valence and conduction bands but also forms energy states within the band gap, leading to a smaller band gap.

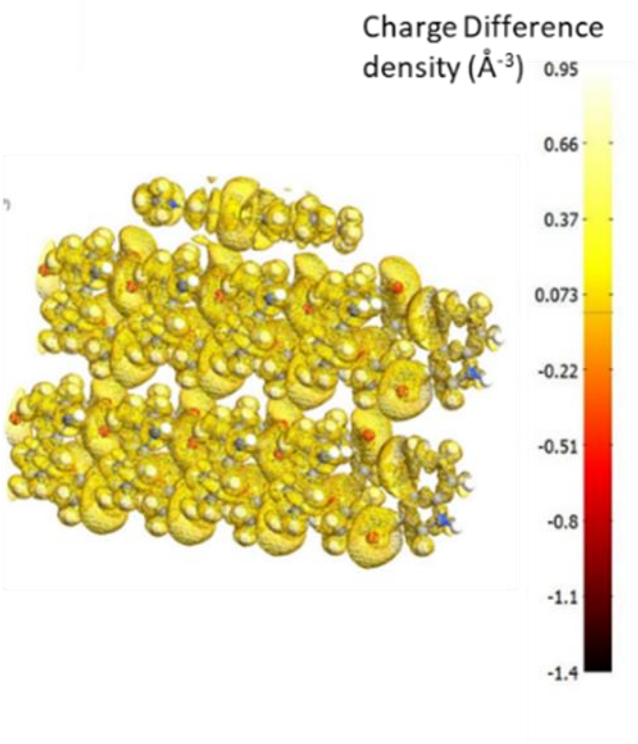

Fig S8. Electron charge density difference map for Cyclo-Gly-Trp with adsorbed MB. This map shows changes in electron density distribution following the interaction of the peptide and MB. Analysis of the map suggests that the charge density isosurface shows a relatively large overlap between charge densities of MB and the peptide, indicating the existence of an electrostatic interaction. The molecular orbital charge densities are concentrated around the indole ring of the tryptophan moiety in Cyclo-Gly-Trp. This arrangement facilitates charge transfer via the aromatic networks within the peptide.

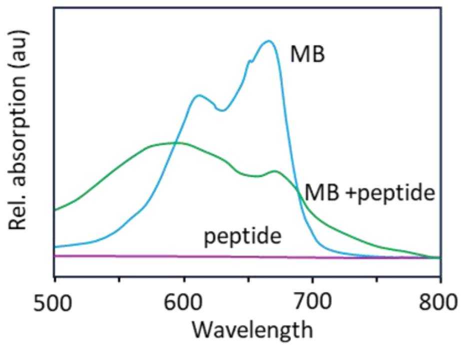

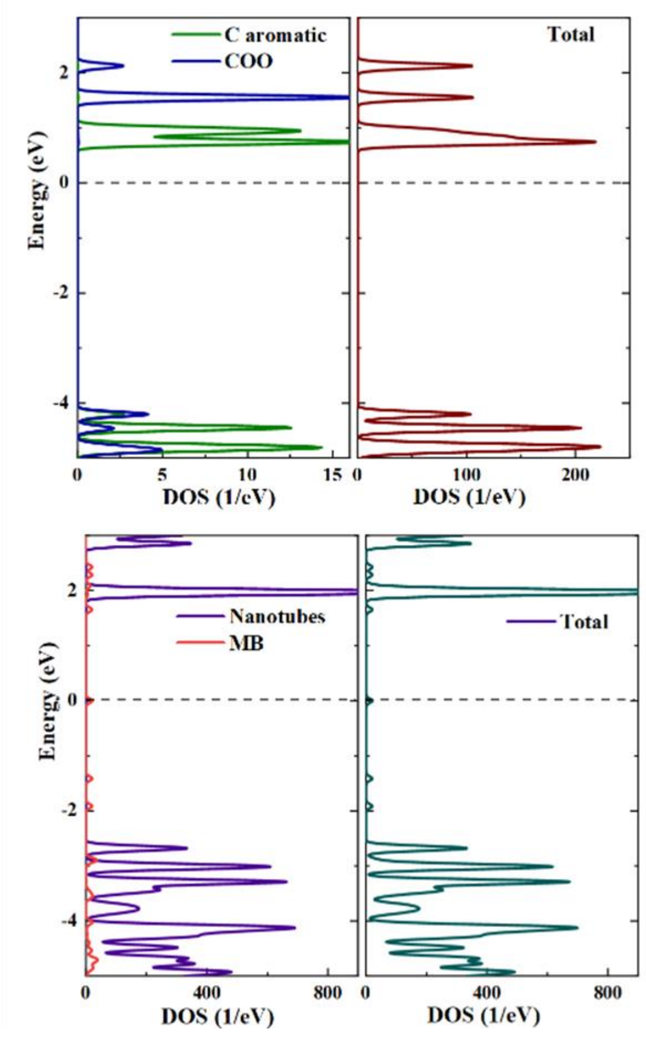

S9. (left) Optical absorption spectra of Phe-Phe, MB, and then combined. (right) Calculated total and projected density of states (DOS) for (top) pristine Phe-Phe and (below) MB on Phe-Phe, showing only the 2p states.

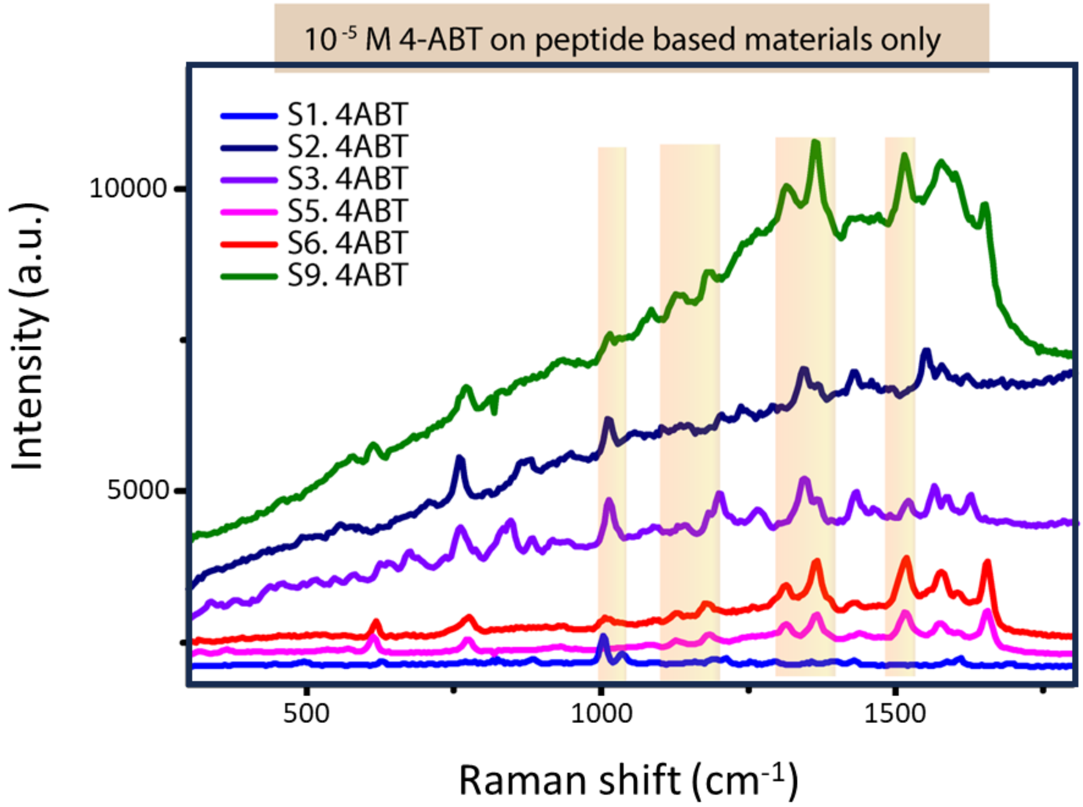

Fig S10. Peptide semiconductor-enhanced Raman scattering (PSERS) using 4ABT (4-aminothiophenol), for a range of peptides at a concentration of 10^{-5} M. The yellow bars show the spectral region associated with the Raman bands for the probe molecule.

Section 2 Materials and methods

Template formation and oven heating

Peptide nanotubes were formed by dissolving peptide in 1,1,1,3,3,3-hexafluoro-2-propanol (Sigma-Aldrich) to a concentration of 100 mg/ml, which was further diluted in deionized water to a final concentration of 2 mg/ml. Peptides were purchased from Bachem at purity >99%.

Probe molecule solutions

To prepare meso-Tetra (N-methyl-4-pyridyl) porphine tetrachloride (TMPyP; T40125, Frontier Scientific) solutions, TMPyP powder was diluted with deionized water to a range of concentrations from 10^{-4} to 10^{-7} M. Methanol blue (MB), thymine, cytosine, and uracil were all sourced from Sigma-Aldrich, Ireland and similarly diluted with deionized water to a range of concentrations from 10^{-4} to 10^{-7} M.

Crystal violet 1% aqueous solution (CAS Number: 548-62-9) was diluted in distilled water down to the concentration of 10^{-5} M. Methyl Green (zinc chloride salt, ~85%; CAS:7114-03-6) was dissolved in distilled water at initial concentration 10^{-2} M and then diluted down to 10^{-5} M using deionized water. Methyl Red (ACS reagent, crystalline; CAS Number 493-52-7) was prepared in chloroform (analytical reagent grade; CAS: 67-66-3) at initial concentration 10^{-2} M and then diluted down to 10^{-5} M using chloroform. The probe molecule solutions were drop cast (40 µl) on the pristine and annealed NTs and left to dry for 2-3 hours prior to Raman measurements.

Characterization

Optical absorbance measurements (UV-vis)

The UV-vis spectra were recorded (V-650, JASCO, Inc.) over a 190–900 nm wavelength range. UV-vis was undertaken using a 1 nm step size, with a 1 nm bandwidth, and a 400 nm/min scan speed. Sample preparation for UV-vis was as described above. Briefly, the NTs on glass coverslips annealed at different temperatures were prepared for UV-vis measurements to be undertaken, with and without the probe molecules.

Circular Dichroism (CD) Spectroscopy. Circular dichroism

(CD) spectra were obtained with a Chirascan CD spectrometer (Applied Photophysics, U.K.). The wavelengths from 190 to 250 nm were scanned every 4 s.

Fourier transform infrared (FTIR) spectroscopy

FTIR spectra were recorded using transmission and absorption modes (Alpha.Platinum-ATR, Bruker) using the same sample preparation as for UV-vis.

Contact angle measurements

A contact angle measuring system (DSA10, Krüss) equipped with a camera was used to measure contact angles of 10 µl droplets of deionized water placed on each sample.

Scanning electron microscopy (SEM) and energy-dispersive X-ray spectroscopy (EDX) mapping

Scanning electron microscopy (JSM-7600F, JEOL, operated at 5 kV) was employed to characterize the pristine and annealed NTs. A thin (~8 nm) layer of gold was sputtered on the samples before SEM imaging (Hummer IV, Anatech USA). EDX mapping was performed using the same system at 10 kV and 10 nA.

X-ray photoelectron spectroscopy

The samples were analyzed by X-ray photoelectron spectroscopy (XPS) (Axis Ultra^{DLD}, Kratos, Ltd., UK) using an Al K_α (1486.7 eV) X-ray source. All samples were outgassed for 12 h under ultra-high vacuum before analysis.

Raman and surface enhanced Raman spectroscopy

In order to record SERS spectra, a bespoke Raman system was employed. This system consists of an inverted optical microscope (IX71) with a SP-2300i spectrograph (Princeton Instruments), and an EMCCD camera (IXON). The Raman excitation wavelength was 532 nm, which was fixed at ~5 mW incident laser power. The same system was used for photoluminescence measurements. The CCD camera was calibrated over the spectral window using toluene. Each spectrum shown is an average of spectra recorded from ten different spots on a sample unless otherwise stated. SERS enhancement factor values were calculated by comparing the intensity of the appropriate MB peak (at 1560 cm^{-1}) and TMPyP (at 1535 cm^{-1}) MR peak at 1395 cm, CV 1616 cm^{-1}, Mg 1621 cm^{-1} measured in the SERS experiments to the corresponding peak measured from normal Raman spectra of the probe molecules without the use of NT materials.

The SERS enhancement factor (EF) is given by:

$EF= N_{vol}\ I_{SERS}\ /N_{surf}\ I_{Raman}$

where N_{vol} and N_{surf} are the number of molecules probed in the sample and on the SERS substrates, respectively. I_{SERS} and I_{Raman} are the corresponding SERS intensities and normal Raman. Assuming the number of molecules are the same on the substrate as we are using the same drop size of materials. Both I_{SERS} and I_{Raman} is peak to peak intensity therefore the bassline was subtracted from the initial beak intensity. The EF was calculated several times each time resulting in similar EF for each SERS substrate.

Kelvin probe measurements

Kelvin Probe Force Microscopy (KPFM) measurements were carried out to investigate the Fermi energy in self-assembled peptide nanofibers-like structures. Each peptide type was studied individually via contact potential measurements. AFM/KPFM measurements were conducted at ambient conditions. The microscope has a closed feedback loop. The FM-KPFM mode uses the frequency shift of the cantilever oscillation to detect the electrostatic force gradient. As the tip was grounded during the KPFM measurements, the contact potential difference (V_{CPD}) is determined by $V_{CPD} = \varphi_s - \varphi_{tip}/|e|$, with e being the elementary charge and φ_s and φ_{tip} are the work functions of the sample and tip, respectively. The work function φ_s can be expressed as a function of the position of E_{VAC} and the Fermi level of the material.

Theoretical calculations

We performed our first principles computation using the local combination of the atomic orbitals (LCAO) methodology and density function theory (DFT) as it is implemented in the QuantumATK program [2]. The exchange-correlation functional utilized in this study was based on the Perdew, Burke, Ernzerhof (PBE) [3] related to the generalized gradient approximation (GGA). The norm-conserving FHI pseudopotentials were employed with a double-ζ polarized (DZP) set and an 75 Ha density mesh cutoff energy. The Monkhorst-Pack special k-point grid of $1 \times 1 \times 4$ for structural optimization and $1 \times 1 \times 7$ for electronic characteristic calculations of Cyclo-Gly-Trp nanowire-like and MB on Cyclo-Gly-Trp nanowire-like (MB on Phe-Phe) was used to execute the Brillouin region. When calculating the self-consistent field (SCF), the energy convergence tolerance limit of 10^{-6} Ha was taken into account. Including the force on each atom less than 0.05 eV/ Å, the geometry frameworks were performed employing the limited-memory Broyden–Fletcher–Goldfarb–Shanno (LBFGS) method.

2. S. Smidstrup, T. Markussen, P. Vancraeyveld, J. Wellendorff, J. Schneider, T. Gunst, B. Verstichel, D. Stradi, P.A. Khomyakov, U.G. Vej-Hansen, M.E. Lee, S.T. Chill, F. Rasmussen, G. Penazzi, F. Corsetti, A. Ojanperä, K. Jensen, M.L.N. Palsgaard, U. Martinez, A. Blom, M. Brandbyge, K. Stokbro, QuantumATK: An integrated platform of electronic and atomic-scale modelling tools, J. Phys. Condens. Matter. 32 (2020) 015901.
3. P. Perdew, K. Burke, M. Ernzerhof, Generalized gradient approximation made simple, Phys. Rev. Lett. 77 (1996) 3865–3868.
